# Hepatoprotective Role of Carvedilol against Ischemic Hepatitis Associated with Acute Heart Failure via Targeting miRNA-17 and Mitochondrial Dynamics-Related Proteins: An In Vivo and In Silico Study

**DOI:** 10.3390/ph15070832

**Published:** 2022-07-05

**Authors:** Doaa I. Mohamed, Samar F. Ezzat, Wael M. Elayat, Omnyah A. El-Kharashi, Hanaa F. Abd El-Kareem, Hebatallah H. Abo Nahas, Basel A. Abdel-Wahab, Samar Zuhair Alshawwa, Asmaa Saleh, Yosra A. Helmy, Eman Khairy, Essa M. Saied

**Affiliations:** 1 Department of Clinical Pharmacology and Therapeutics, Faculty of Medicine, Ain Shams University, Cairo 11566, Egypt; omnyah_aly@med.asu.edu.eg; 2 Department of Histology and Cell Biology, Faculty of Medicine, Ain Shams University, Cairo 11566, Egypt; samarezzat@med.asu.edu.eg; 3 Department of Medical Biochemistry and Molecular Biology, Faculty of Medicine, Ain Shams University, Cairo 11566, Egypt; wael_elayat@med.asu.edu.eg (W.M.E.); dreman_khairy@med.asu.edu.eg (E.K.); 4 Zoology Department, Faculty of Science, Ain Shams University, Abbasseya, Cairo 11566, Egypt; hanaafathy@sci.asu.edu.eg; 5 Physiology Department, Faculty of Science, Suez Canal University, Ismailia 41522, Egypt; hebatallah_hassan@science.suez.edu.eg; 6 Department of Medical Pharmacology, College of Medicine, Assiut University, Assiut 71515, Egypt; basel_post@msn.com; 7 Department of Pharmacology, College of Pharmacy, Najran University, P.O. Box 1988, Najran 55461, Saudi Arabia; 8 Department of Pharmaceutical Sciences, College of Pharmacy, Princess Nourah Bint Abdulrahman University, P.O. Box 84428, Riyadh 11671, Saudi Arabia; szalshawwa@pnu.edu.sa (S.Z.A.); asali@pnu.edu.sa (A.S.); 9 Department of Animal Hygiene, Zoonoses and Animal Ethology, Faculty of Veterinary Medicine, Suez Canal University, Ismailia 41522, Egypt; yosra_helmy@vet.suez.edu.eg; 10 Department of Veterinary Science, University of Kentucky, Lexington, KY 40504, USA; 11 Chemistry Department, Faculty of Science, Suez Canal University, Ismailia 41522, Egypt; 12 Institute for Chemistry, Humboldt Universität zu Berlin, Brook-Taylor-Str. 2, 12489 Berlin, Germany

**Keywords:** ischemic hepatitis, acute heart failure, carvedilol, histopathology, oxidative stress, MAPK, PGC-1α, DNM1L, mitofusin 2, miRNA-17, molecular modelling

## Abstract

Acute heart failure (AHF) is one of the most common diseases in old age that can lead to mortality. Systemic hypoperfusion is associated with hepatic ischemia–reperfusion injury, which may be irreversible. Ischemic hepatitis due to AHF has been linked to the pathogenesis of liver damage. In the present study, we extensively investigated the role of mitochondrial dynamics-related proteins and their epigenetic regulation in ischemic liver injury following AHF and explored the possible hepatoprotective role of carvedilol. The biochemical analysis revealed that the ischemic liver injury following AHF significantly elevated the activity of alanine aminotransferase (ALT), aspartate aminotransferase (AST), and alkaline phosphatase (ALP) enzymes, the level of total and direct bilirubin, and the expression of hepatic mitogen-activated protein kinase (MAPK), dynamin-1-like protein (DNM1L), and hepatic miRNA-17. At the same time, it significantly reduced the serum albumin level, the activity of hepatic superoxide dismutase (SOD), and the expression of mitochondrial peroxisome proliferator-activated receptor-1α (PGC-1α), and mitofusin 2 (Mtf2). The histological examination of the liver tissue revealed degenerated hepatocytes. Interestingly, administration of carvedilol either prior to or after isoprenaline-induced AHF significantly improved the liver function and reversed the deterioration effect of AHF-induced ischemic hepatitis, as demonstrated by biochemical, immunohistochemical, and histological analysis. Our results indicated that the hepatoprotective effect of carvedilol in ameliorating hepatic ischemic damage could be attributed to its ability to target the mitochondrial dynamics-related proteins (Mtf2, DNM1L and PGC-1α), but also their epigenetic regulator miRNA-17. To further explore the mode of action of carvedilol, we have investigated, in silico, the ability of carvedilol to target dynamin-1-like protein and mitochondrial dynamics protein (MID51). Our results revealed that carvedilol has a high binding affinity (−14.83 kcal/mol) toward the binding pocket of DNM1L protein. In conclusion, our study highlights the hepatoprotective pharmacological application of carvedilol to attenuate ischemic hepatitis associated with AHF.

## 1. Introduction

Acute heart failure (AHF) is a severe public health problem that negatively influences people’s quality of life and shortens their longevity. Cardiac output is considered as a product of heart rate by stroke volume and depends on the heart and the circulatory system’s veins and arteries. The cardiac output is elevated during physiologic stress to ensure adequate tissue perfusion [1]. AHF is defined as the lack of systemic perfusion to fulfil the body’s metabolic demands. It is often caused by ventricular pump malfunction but may infrequently appear with symptoms of a noncardiac condition such as hepatic dysfunction [2]. The liver receives approximately 25% of the cardiac output, so it is more susceptible to hypoperfusion upon reduced cardiac output since the hepatic blood flow is reduced [3]. Under hepatocellular damage, a variety of liver enzymes, including serum alanine aminotransferase (ALT), aspartate aminotransferase (AST), and other biomarkers of liver function (e.g., alkaline phosphatase (ALP) level) are raised considerably, by two to three times compared to the normal range [4]. Additionally, the total bilirubin level is elevated up to 3 mg/dL, and the albumin synthesis may be impaired, resulting in hypoalbuminemia and an increase in fluid accumulation [2].

In AHF, the hepatic ischemia–reperfusion injury is characterized by the early activation of Kupffer cells, the elevation of intracellular calcium, oxidative stress, mitochondrial damage, and disruption of the liver microcirculation [5]. When the liver is subjected to AHF, reactive oxygen species (ROS) are generated and may represent pivotal mediators of the ensuing pathological complications [5]. In chronic hypoperfusion, the hepatocytes suffer from low blood flow and oxygen levels, leading to impaired mitochondrial functions, elevated ROS levels, hypoxia, and necrosis [6]. Hepatocytes can tolerate mild oxidative stress through different antioxidant defense mechanisms, including superoxide dismutase (SOD), recognizing and removing oxidized molecules [7]. SOD is an enzymatic antioxidant present in the cytosol and in the space between the inner and outer mitochondrial membranes and is responsible for preventing ROS-induced toxicity [8].

Mitochondria are the gatekeepers of cell death and contribute to a wide range of cellular mechanisms such as ATP synthesis, inflammation, and cellular metabolism [9]. Mitochondrial electron transport is an enzymatic source of ROS generation and also a target of oxidant-induced damage [10]. Prolonged increases in ROS production in the mitochondria can lead to a catastrophic cycle of mitochondrial DNA damage, radical oxygen generation, and cellular injury [11]. In response to pathophysiologic stimuli, the mitochondrial dynamics network maintains the fusion and fission equilibrium, interlinked mechanisms that coordinate appropriate mitochondrial activities [12]. The mitochondrial dynamics are associated with the translocation of the protein to the inner or outer mitochondrial membranes [12].

Mitochondria can change their morphology to meditate the equilibrium between fission and fusion activities, which is critical for cell health [13]. The role of mitochondrial fusion is to repair the damaged elements which are produced during ROS detoxification and modulate the mitochondrial form, size, quantity, and bioenergetic activity [14]. Mitofusins (Mtf1/2) and dynamin-1-like protein (DNM1L) (also called dynamin-related protein 1 (DRP1)) are regarded as the most critical proteins mediating the mitochondrial fusion and fission equilibrium [15]. Mitofusin 2 (Mtf2) is extensively produced in the heart, where it is found at the exterior membrane of the mitochondria to regulate mitochondrial fusion [15]. The binding of mitochondria with Mtf2 is necessary for the fusion of the exterior membrane of the mitochondria. Diminishing Mtf2 expression leads to a failure of fusion and mitochondrial loss [16]. DNM1L is a cytosolic dynamin-related GTPase protein involved in mitochondrial fission by mediating constriction of the mitochondrial membrane [15].

In acute hepatic hypoxia, a drop in intracellular ATP levels stimulates mitochondrial fission to maintain mitochondrial volume. The balance between fission and fusion activities is considered as a cell defense mechanism against oxidative damage and it protects against apoptotic stimuli [17]. Interruption of the mitochondrial fission/fusion equilibrium results in mitochondrial disintegration and stimulation of the apoptosis pathway, such as the mitogen-activated protein kinase (MAPK) pathway. The MAPK pathway is a well-known regulator of hepatic metabolism and disorder [18]. Further, MAPK regulates gluconeogenesis by triggering the peroxisome proliferator-activated receptor co-activator 1α (PGC-1α) [19]. PGC-1α is a significant regulator of mitochondrial biogenesis, ROS metabolism, transcription of several genes, respiration, and gluconeogenesis [20]. PGC-1α stimulates mitochondrial biogenesis by triggering multiple transcription factors and upregulating the expression of nuclear and mitochondrial proteins that regulates thermogenesis in brown adipose tissue, lipid metabolism, and liver gluconeogenesis [20,21]. Interestingly, it was found that the expression of PGC-1α and Mtf2 proteins are down-regulated under hepatic hypoperfusion [22].

Since the exact mechanism of liver injury is not fully understood, the therapeutic modalities and/or prophylactic measures are still not satisfactory. This knowledge gap encouraged us to envision and explore the relationship between acute hypoperfusion induced by AHF and mitochondrial dynamics. The mechanisms that contribute to the pathogenesis of hepatic ischemia replication include endoplasmic reticulum stress, mitochondrial dysfunction, oxidative stress, inflammation, and apoptosis; these incidences are also influenced by MicroRNAs (miRNAs) [23]. The miRNAs possess a broad range of modulatory effects on genetic expression [24]. Among miRNA, the miRNA-17 gene cluster is encoded on chromosome 13 and is essential for tissue development. The miRNA-17 is a pro-inflammatory miRNA that plays a crucial role in liver fibrosis [25]. Emerging research has shown that miRNA-17 plays a role in a variety of hepatic pathophysiology by mediating autophagy [25]. Upregulation of miRNA-17 in hepatic reperfusion reduced the expression of signal transducer and activator of transcription 3 (STAT3) and phosphorylated STAT3 (p-STAT3), which stimulates autophagy in response to ischemia–reperfusion. It is worth noting that STAT3, an essential cellular survival factor, has an anti-apoptotic role in response to oxidative stress and seems to be associated with miRNA-17 [26]. It was recently documented that mitochondrial fusion-related Mtf1 and Mtf2 expression is related to miRNA-17 expression in diabetes mellitus [27].

**Figure 1:**
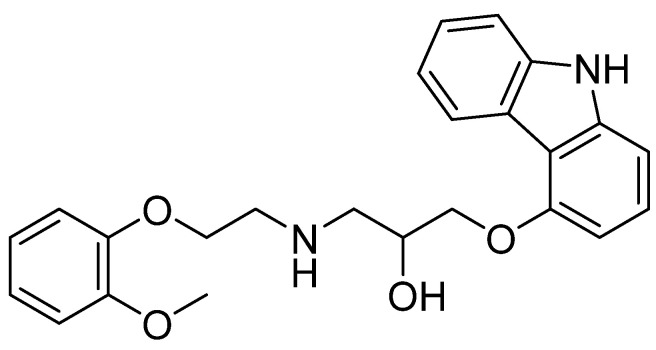
Chemical structure of carvedilol.

The β-blocker drugs are commonly used for hypertension management to diminish left ventricular hypertrophy [28]. They are also helpful for patients with AHF to reduce stress responses [29]. Among the β-blocker drugs, carvedilol is a 3rd generation vasodilator β-blocker used to treat hypertension (Figure 1) [29]. Carvedilol is a potent β-adrenergic receptor antagonist with antioxidant properties, which inhibit the mitochondrial permeability transition [30]. Carvedilol is effective only when the mitochondrial permeability transition is triggered by a primary oxidative process [30]. The cardioprotective benefits of carvedilol in ischemia–reperfusion injury most likely rely on a combination of different pathways, including adrenoreceptor inhibition, vasodilation, and antioxidant capacity [30]. Furthermore, carvedilol has antioxidant properties that prevent ROS formation in the myocardium and suppress the free radical-induced triggering of transcription factors and apoptosis [31]. It also suppresses the expression of several genes involved in myocardial injury and cardiac remodeling [32]. These findings suggest that carvedilol could be applied to treat ischemic hepatitis associated with AHF by targeting the mitochondrial dynamics network.

Encouraged by these facts, we aimed to investigate the role of mitochondrial dynamics-related proteins and their epigenetic regulator miRNA-17 in ischemic hepatitis associated with acute heart failure. Further, we explored the possible hepatoprotective effect of carvedilol by targeting this pathway via biochemical, histological, and molecular docking studies.

## 2. Results

### 2.1. Biochemical Analysis

#### 2.1.1. Effect of Hepatic Ischemia Associated with AHF on Liver Function and Assessment of Carvedilol Administration

First, we investigated the effect of isoprenaline-induced AHF on liver function by assessing AST, ALT, ALP, total bilirubin, direct bilirubin, and albumin. As shown in Table 1, isoprenaline administration induced a significant (*p* < 0.05) upregulation in AST and ALT activities with an AST/ALT ratio elevation. At the same time, it caused a significant (*p* < 0.05) increase in the total and direct bilirubin and ALP, while it significantly reduced (*p* < 0.05) serum albumin. These results indicate that isoprenaline-induced AHF impaired hepatic function in a pattern that has typically been consistent with cholestasis and primary hepatocellular injury. These findings may be attributed to impaired blood flow, liver congestion, and oxidative stress. In order to explore the effect of carvedilol administration on AHF-induced hepatic ischemia, we have assessed the effect of carvedilol treatment on hepatic function before and after isoprenaline-induced AHF. Interestingly, administration of carvedilol either before or after isoprenaline-induced AHF significantly improved and reversed the hepatic function to almost that of the control group. As indicated in Table 1, treatment with carvedilol (30 mg/kg) significantly decreased the liver enzyme activity (AST, ALT, ALP) and the total and direct bilirubin, while it significantly (*p* < 0.05) increased the serum albumin. These results may indicate that carvedilol has the capability to restore hepatic function and activity to normal conditions and prevent the hepatic ischemia associated with AHF by amelioration of oxidative stress and the subsequent hepatocellular injury.

#### 2.1.2. Effect of AHF-Induced Hepatic Ischemia on the Expression of Oxidative Stress Markers and Influence of Carvedilol Administration

**Figure 2:**
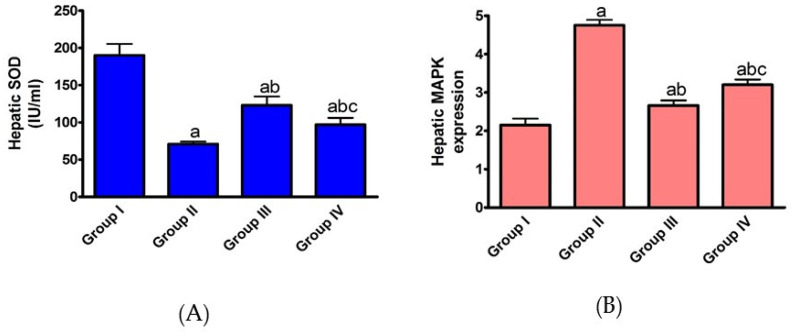
Effect of carvedilol on hepatic (**A**) SOD and (**B**) MAPK expression in hepatic ischemia-associated isoprenaline-induced AHF in male Westar rats. Group I: naïve group, Group II: control positive, Group III: carvedilol pre-treated, Group IV: carvedilol post-treated. Data presented as mean ± SEM. ^a^ *p* < 0.05 versus group I, ^b^ *p* < 0.05 versus group II, ^c^ *p* < 0.05 versus group III.

In order to explore the effect of AHF-induced hepatic ischemia on oxidative stress markers, the expression of SOD and MAPK was evaluated. As indicated in Figure 2, isoprenaline administration induced a significant (*p* < 0.05) decrease in SOD expression, while it significantly increased MAPK expression. These results indicate that hepatic ischemia induces upregulation of ROS production and overexpression of MAPK. Under excess ROS production, the endogenous antioxidant defense system devastates (such as, SOD), and the cell experiences oxidative stress and damage.

On the other hand, pre- and post-administration of carvedilol caused a significant (*p* < 0.05) increase in SOD, while it induced a significant (*p* < 0.05) decrease in the expression of MAPK. This may be explained by the effect of carvedilol on lipid peroxidation in CHF patients by functioning as a free radical scavenger. Carvedilol may also decrease neutrophil accumulation by decreasing intracellular adhesion molecules, promoting neutrophil attachment to endothelial and smooth muscle cells and inhibiting ROS generation. Our results indicate that the hepatoprotective potency of carvedilol could rely on its ability to modulate the oxidative stress biomarkers under AHF-induced hepatic ischemia.

#### 2.1.3. Effect of Carvedilol Treatment on the Expression of PGC-1α, Mitofusin 2, and DNM1L Proteins in Hepatic Ischemia Associated with AHF

**Figure 3:**
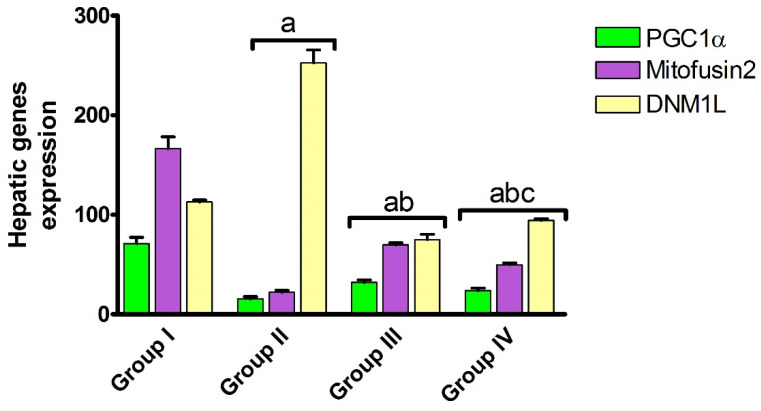
Effect of carvedilol on hepatic PGC-1α, mitofusin 2, and DNM1L expression in hepatic ischemia associated with isoprenaline-induced AHF. Group I: naïve, Group II: control positive, Group III: pre-treated carvedilol, Group IV: post-treated carvedilol. Data presented as mean ± SEM. ^a^ *p* < 0.05 versus group I, ^b^ *p* < 0.05 versus group II, ^c^ *p* < 0.05 versus group III.

Mitochondrial morphology is modulated by fusion and fission dynamics. The hepatic Mtf2 and DNM1L proteins contribute to modulating the continuous division and fusion of mitochondria, which is essential to achieve the mitochondrial biological function. PGC-1α is a transcriptional coactivator controlled by the MAPK family and has a potent hepatoprotective activity by amplifying ROS-detoxifying enzymes. To gain insight into the hepatic mitochondrial fission and fusion under AHF-induced hepatic ischemia, the expression level of PGC-1α, mitofusin 2 and DNM1L proteins were assessed. As shown in Figure 3, isoprenaline administration induced a significant (*p* < 0.05) decrease in the expression of PGC-1α and Mtf2, while it significantly (*p* < 0.05) increased the expression of DNM1L. Our results revealed that, under AHF-induced hepatic ischemia, the hepatic mitochondrial fusion is dysregulated, and the mitochondria biogenesis is downregulated with an increase in ROS production and an overall reduction in mitochondrial function.

Again, administration of carvedilol either before or after isoprenaline treatment significantly (*p* < 0.05) increased the expression of PGC-1α and Mtf2 proteins, while it significantly (*p* < 0.05) reduced DNM1L expression, with preferable effects toward the prophylactic regimen. Carvedilol, as a ROS scavenger, might upregulate the expression of antioxidant enzymes, which protect the mitochondria from oxidative damage. Further, it might reduce ROS-induced ischemia by improving cardiac function and hepatic blood supply, and decreasing hepatic congestion. These results indicate that carvedilol has the ability to trigger mitochondrial biogenesis by the upregulation of PGC-1α and mitochondrial Mtf2. The impact of carvedilol on DNM1L and mitofusin 2 expression is unique to our study and it may depend on its antioxidant effect or its effect on epigenetic regulation mechanisms.

#### 2.1.4. Effect of AHF-Induced Hepatic Ischemia on miRNA-17 Expression and Assessment of Carvedilol Administration

**Figure 4:**
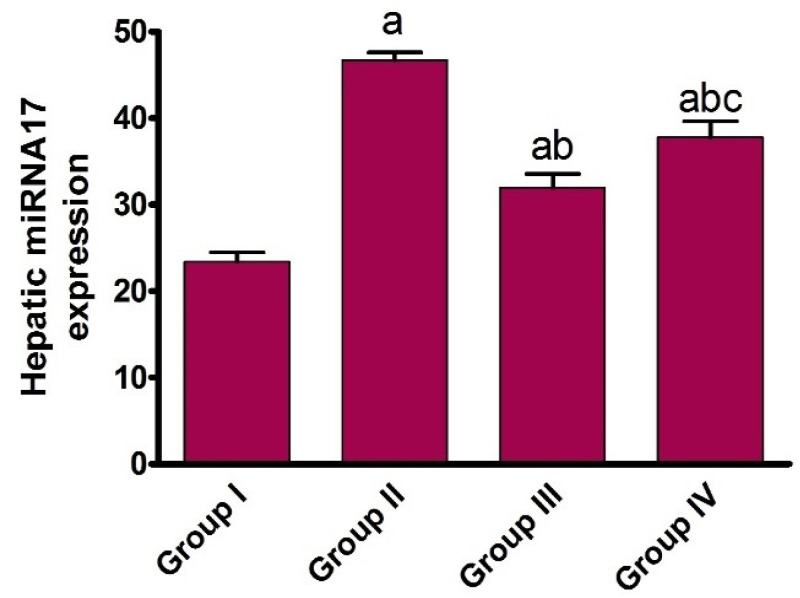
Effect of carvedilol on miRNA-17 expression in hepatic ischemia associated with isoprenaline-induced AHF. Group I: naïve, Group II: control positive, Group III: pre-treated carvedilol, Group IV: post-treated carvedilol. Data presented as mean ± SEM. ^a^ *p* < 0.05 versus group I, ^b^ *p* < 0.05 versus group II, ^c^ *p* < 0.05 versus group III.

MiRNAs have a role in a variety of hepatic pathological processes via regulating hepatic mitochondria formation [26]. In our study, we have first defined the list of regulatory and associated miRNA genes which play a role in mitochondrial dynamics modulation, utilizing Mtf2 and MAPKs as inputs in our analysis. The results revealed that the pathway enrichment analysis of selected miRNA-17 has a higher number of target genes. Recent data showed that the upregulation of miRNA-17 expression induces hepatic autophagy in hepatic ischemia–reperfusion injury via the amplification of microtubule-associated protein 1 light B II and downregulation of signal transducer and activator of transcription 3 (STAT3) and phosphorylated STAT3 levels [25]. However, the correlation between miRNA-17 and the molecular epigenetic regulation of mitochondrial biogenesis during hepatic ischemia is still obscure. To gain insights into this correlation, expression of miRNA-17 in liver tissue was assessed. As shown in Figure 4, isoprenaline administration induced a significant (*p* < 0.05) increase in the expression of miRNA-17. Previous studies showed that miRNA-17 levels significantly upregulate in hepatic ischemia/reperfusion which leads to a reduction in hepatic cell viability. Our findings may indicate that the high expression of miRNA-17 could be the promotion factor of hepatic ischemia by modulating Mtf2 expression.

Interestingly, the administration of carvedilol either before or after isoprenaline treatment significantly (*p* < 0.05) diminished the expression of miRNA-17, with preferable effects toward the prophylactic regimen. These results indicate that the protective effect of carvedilol could be attributed to its ability to target the expression of miRNA-17. To the best of our knowledge, this is the first report to examine the effect of carvedilol on the expression of miRNA-17. The impact of carvedilol in targeting the expression of Mtf2 may be explained by its influence on the expression of miRNA-17.

### 2.2. Histological Analysis

#### 2.2.1. Light Microscopic Analysis

To confirm our biochemical findings, the effect of AHF-induced hepatic ischemia and the possible protective effect of carvedilol have been explored using light microscopic examination of H&E-stained sections of the liver tissue, in addition to the determination of liver injury by calculating the number and frequency distribution of each component in four different field sections in five rats (20 fields/group) (Table 2).

**Figure 5:**
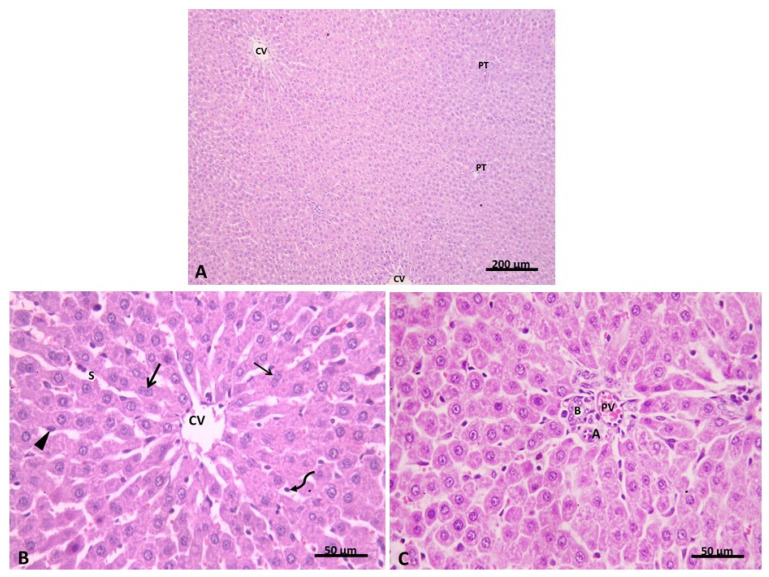
(**A**) showing the regular appearance of classic hepatic lobules with central veins (CV) and peripheral portal tracts (PT). (**B**) Group I. H&E ×100 shows hepatocyte cords radiating from the central vein (CV). Polygonal hepatocytes are seen with acidophilic cytoplasm containing basophilic granules and central rounded vesicular nuclei. Some cells are binucleated (↑). Blood sinusoids (S) are lined with flat endothelial cells (▲) and Kupffer cells (curved arrow). (**C**) Group I. H&E × 400 showing portal tract containing branches from the portal vein (PV), hepatic artery (A), and bile duct (B) Group I. H&E ×400.

The stained liver section of the control group showed classic hepatic lobules. They were seen with central veins (CVs) and peripherally situated portal tracts. Branching and anastomosing cords of hepatocytes were seen radiating from the CV. Hepatocytes appeared polygonal in shape with acidophilic granular cytoplasm and central, rounded, vesicular nuclei (Figure 5A–C). Further, we verified the extent of liver injury (Table 2). We found that 90% of sections had no liver congestion, while 10% of these sections had minimal congestion. Additionally, 95% of sections had no vacuolization, and 5% had minimal vacuolization. There were no signs of necrosis in the hepatic sections of the control group.

**Figure 6:**
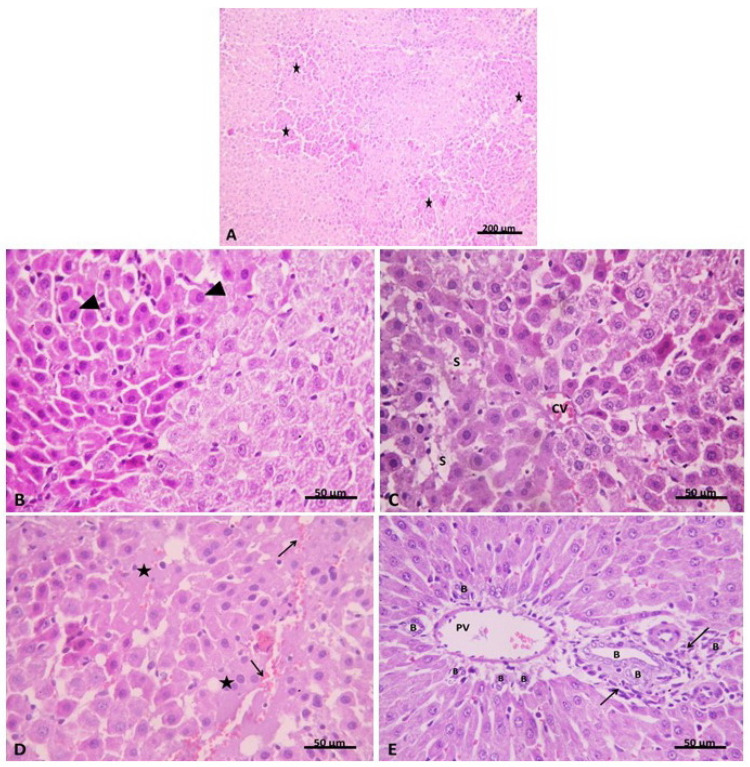
(**A**) showing disruption of the normal hepatic architecture with focal areas of deeply stained cells (*). (**B**) Group II. H&E x100 showing the focal area of hepatocytes with deep acidophilic cytoplasm and small dark nuclei pushed peripherally in some cells (▲). (**C**) Group II. H&E ×400 showing congested central vein (CV). The radiating hepatocytes appeared with deep acidophilic cytoplasm and small dark nuclei. Notice the dilated blood sinusoids (S). (**D**) Group II. H&E ×400 showing areas of hemorrhage (↑) and areas of homogenous acidophilic material (*) between hepatocytes. (**E**) Group II. H&E ×400 showing an expanded portal tract with the dilated portal vein (PV) and proliferation of the bile ducts (B). Mononuclear cellular infiltration is also evident (↑). Group II. H&E ×400.

In the isoprenaline-treated group (Group II), disruption of the normal hepatic architecture was evident with focal areas of deeply stained cells (Figure 6A). Hepatocytes in these areas were seen with deeply stained acidophilic cytoplasm and small dark nuclei which were pushed peripherally in some cells. Hemorrhage and homogeneous pale acidophilic areas were also noticed in the hepatic lobules (Figure 6B). The affected hepatocytes were mainly seen around the congested central vein (centrilobular) (zone I) of the hepatic lobule, while other hepatocytes in zones II and III appeared with acidophilic cytoplasm and vesicular nuclei. Blood sinusoids appeared dilated and congested in some areas (Figure 6C). Portal tracts appeared expanded with dilation, congestion of portal vein branches and proliferation of bile ducts. Mononuclear cellular infiltration was also evident (Figure 6D). Evaluation of the liver injury extent revealed that 15% of isoprenaline hepatic sections had no liver congestion, while 50%, 30% and 5% of these sections had minimal, slight, and moderate congestion, respectively (Table 2). In addition, 67% of sections had no vacuolization and 33% had minimal vacuolization. 10% of sections showed no signs of necrosis; nevertheless, 25% and 65% of sections showed necrosis of individual cells and less than 30% necrotic cells, respectively.

**Figure 7:**
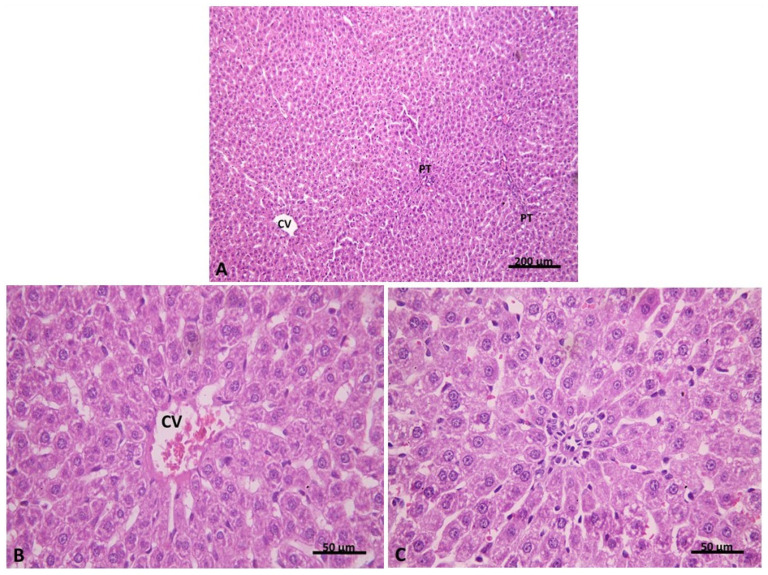
(**A**) showing hepatic architecture more or less similar to the control group with the central vein (CV) and peripheral portal tracts (PT). (**B**) Group III. H&E ×100 showing congested central vein (CV). Hepatocytes are seen with acidophilic cytoplasm and central vesicular nuclei. (**C**) Group IIIa. H&E ×400 showing apparently normal portal area with an apparent decrease in cellular infiltration. Group III. H&E ×400.

In Group III (pre-treatment with carvedilol), hepatic architecture appeared similar to that of the control group (Figure 7A). Most hepatocytes were seen with acidophilic cytoplasm and central vesicular nuclei (Figure 7B). The normal portal area was seen with an apparent decrease in cellular infiltration. (Figure 7C). Liver injury evaluation indicated that 83% of group III hepatic sections had no liver congestion, while 17% of these sections had minimal congestion (Table 2). In addition, 90% of sections had no vacuolization, and 10% had minimal vacuolization. Interestingly, 95% of sections showed no signs of necrosis, but 5% showed necrosis of individual cells. These results indicate that pre-treatment of carvedilol has a protective effect towards AHF-induced hepatic ischemia.

**Figure 8:**
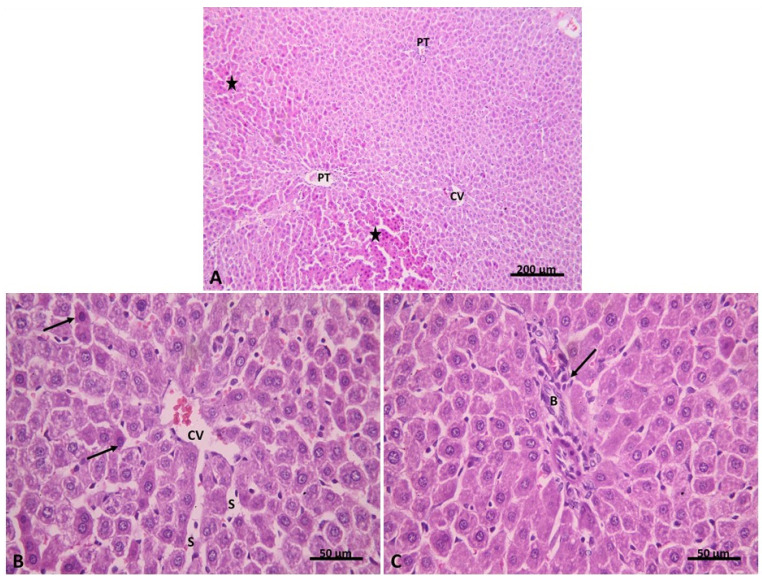
(**A**) showing restoration of the hepatic architecture with few focal areas of deep acidophilic hepatocytes (*). (**B**) Group IV. H&E x100 showing congested central vein (CV). Few hepatocytes are seen with deep acidophilic cytoplasm and small dark nuclei (↑), while the rest of the hepatocytes are seen more or less similar to the control. Blood sinusoids are seen dilated in some areas (S). (**C**) Group IV. H&E ×400 showing portal tract less expanded than group II with an apparent decrease in bile duct (B) proliferation. Cellular infiltration can be seen (↑). Group IV. H&E ×400.

Examination of H&E-stained liver sections of group IV (post-treatment with carvedilol) revealed restoration of the hepatic architecture with few focal areas of affected hepatocytes. Hepatocytes appeared deeply stained, while the remaining hepatocytes looked identical to the control (Figure 8A). Few hepatocytes were seen with deep acidophilic cytoplasm and small dark nuclei around the congested central vein. Blood sinusoids were seen dilated in some areas (Figure 8B). The portal tract appeared less expanded than group II with an apparent decrease in bile duct proliferation and cellular infiltration (Figure 8C). Next, we assessed the grade of liver injury, as indicated in Table 2. The results showed that 50% of group IV hepatic sections had no liver congestion, while 35% and 15% of these sections had minimal and slight congestion. Additionally, 80% of sections had no vacuolization, and 20% had minimal vacuolization. A total of 65% of sections showed no signs of necrosis, but 25% and 10% of sections showed necrosis of individual cells and less than 30% necrotic cells, respectively. These results demonstrate that post-treatment of carvedilol has a protective effect on the hepatic ischemia associated with AHF and has the ability to restore the hepatic architecture distorted by isoprenaline-induced AHF.

#### 2.2.2. Light Microscopic Analysis (Mallory’s Trichrome Stain)

**Figure 9:**
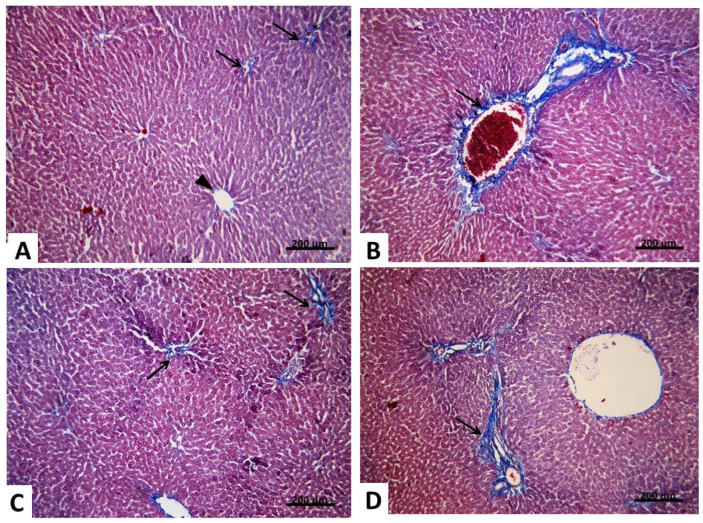
(**A**) Group I. Mallory’s trichrome stain ×250 showing a few collagen fibers (↑) in the portal tract, around the central vein (▲) and in between hepatocytes. (**B**) Group II Mallory’s trichrome stain ×250 showing an apparent increase in the amount of collagen fibers in the portal tract (↑) and around the central vein. (**C**) Group III. Mallory’s trichrome stain ×250 showing collagen fibers (↑) in the portal tract nearly similar to the control group. (**D**) Group IV Mallory’s trichrome stain ×250 showing an apparent decrease in the amount of collagen fibers (↑) in the portal tract and around the central vein.

To further investigate the effect of carvedilol on hepatic ischemia associated with AHF, Mallory’s trichrome stain was performed to assess the degree of fibrous tissue deposition in the liver tissue. In Group I, liver sections stained with Mallory’s trichrome revealed a minimal amount of blue-stained collagen fibers surrounding the central vein and portal areas and in between hepatocytes (Figure 9A). While in Group II, a significant increase in the amount of collagen fibers was noticed around the central vein and in the portal area, as compared to Group I (Figure 9B). In Group III, the amount of collagen fibers seemed to be decreased in the portal tracts and around the central vein, as compared to Group II (Figure 9C). In addition, a significant decrease in the amount of collagen fibers in the portal tract and around the central vein was noticed in Group IV, as compared to Group II (Figure 9D); however, it was significantly increased in comparison to Group I. These results indicated that carvedilol could reduce the fibrous tissue deposition and, thus, ameliorate hepatic cell damage and necrosis. Taken together, these results emphasize the protective effect of carvedilol on liver tissue.

#### 2.2.3. Morphometric and Statistical Results of Mallory’s Trichrome Stain

The mean area percentage of collagen fibers revealed a significant increase (*p* ≤ 0.05) in group II compared with the control group. Meanwhile, there was a significant decrease (*p* ≤ 0.05) in both the carvedilol pre-treated and post-treated groups (III and VI) compared with group II. However, there was significant increase (*p* ≤ 0.05) in the carvedilol post-treated group compared with the control group (Table 3).

#### 2.2.4. Immunohistochemical Analysis

**Figure 10:**
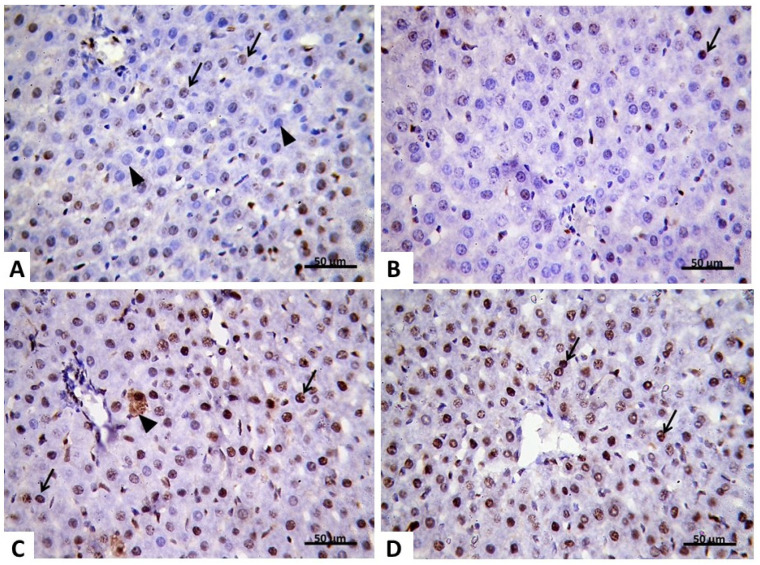
(**A**) Group I showing few hepatocytes with faint PCNA positive nuclei (↑). Notice the negative reaction of other nuclei (▲). (**B**) Group II PCNA ×400 avidin–biotin–peroxidase showing apparent fewer hepatocytes with PCNA positive nuclei (↑). Most hepatocytes show a negative reaction to their nuclei. (**C**) Group III PCNA ×400 avidin–biotin–peroxidase showing few hepatocytes with faint PCNA positive nuclei (↑). Notice the cytoplasmic reaction in some cells (▲). (**D**) Group IV PCNA ×400 avidin–biotin–peroxidase showing most hepatocytes with deep PCNA positive nuclei (↑).

Proliferating cell nuclear antigen (PCNA) is a unique marker for cell proliferation and was first recognized as an antigen generated in cell nuclei during the cell cycle’s DNA synthesis phase. To gain a deep insight into the effect of carvedilol on AHF-induced hepatic ischemia, immunohistochemical study has been performed to evaluate the expression level of PCNA. In the control group, few faint PCNA positive nuclei were seen in the hepatocytes (Figure 10A). In the isoprenaline-treated group (Group II), a significant (*p* < 0.05) decrease in hepatocytes with PCNA positive nuclei was noticed compared with the control group (Table 4). Most hepatocytes showed a negative reaction to their nuclei (Figure 10B). In the pre-treated carvedilol group (Group III), few faint PCNA positive nuclei were seen in the hepatocytes (Figure 10C). While in Group IV, most hepatocytes showed nuclei with an intense positive PCNA immune reaction (Figure 10D). These findings affirm that carvedilol has the capability to ameliorate hepatocyte degeneration in AHF-induced hepatic ischemia by increasing the rate of hepatocyte replication and regeneration.

#### 2.2.5. Morphometric Analysis of PCNA Results

As indicated in Table 4, the results of the morphometric analysis revealed that there was a significant decrease in the mean area percentage of a PCNA positive immune reaction and the mean number of PCNA positive nuclei in Group II compared to the control group. However, a significant increase was noticed in the mean area percentage of the PCNA positive immune reaction and in the mean number of PCNA positive nuclei in Groups III compared to Group II and Group IV (Table 4).

#### 2.2.6. Transmission Electron Microscopic (TEM) Analysis

**Figure 11:**
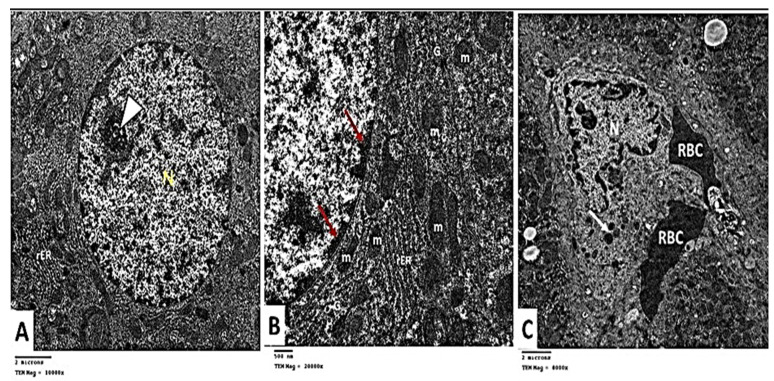
(**A**) Electron micrographs showing hepatocyte with rounded nucleus (N) and prominent nucleolus (▲). Rough endoplasmic reticulum (rER) is seen. Group I TEM × 10,000. (**B**) Electron micrograph showing part of the nucleus with peripheral heterochromatin (↑). Mitochondria (m) with apparent cristae are seen in association with rough endoplasmic reticulum (rER). Glycogen rosettes (G) are seen scattered in the cytoplasm. Group I TEM × 20,000. (**C**) Electron micrograph showing Kupffer cell lining blood sinusoid. Notice the large irregular nucleus (N) and lysosomes (↑) in the cytoplasm. Group I TEM × 8000.

To affirm our histological findings, extensive TEM investigations of hepatocytes have been performed to acquire a deeper understanding of the putative protective impact of carvedilol on hepatic tissue following isoprenaline-induced AHF. TEM analysis of the control group indicated that hepatocytes has central rounded euchromatic nuclei surrounded by a nuclear membrane and had a prominent nucleolus (Figure 11A). The cytoplasm exhibited several mitochondria with cristae variable in shape and size. The mitochondria appeared associated with a rough endoplasmic reticulum (rER). The cytoplasm displayed scattered rosettes of glycogen granules (Figure 11B). Kupffer cells (KCs) lining blood sinusoid were seen. They appeared with a large irregular nucleus and lysosomes in their cytoplasm (Figure 11C).

**Figure 12:**
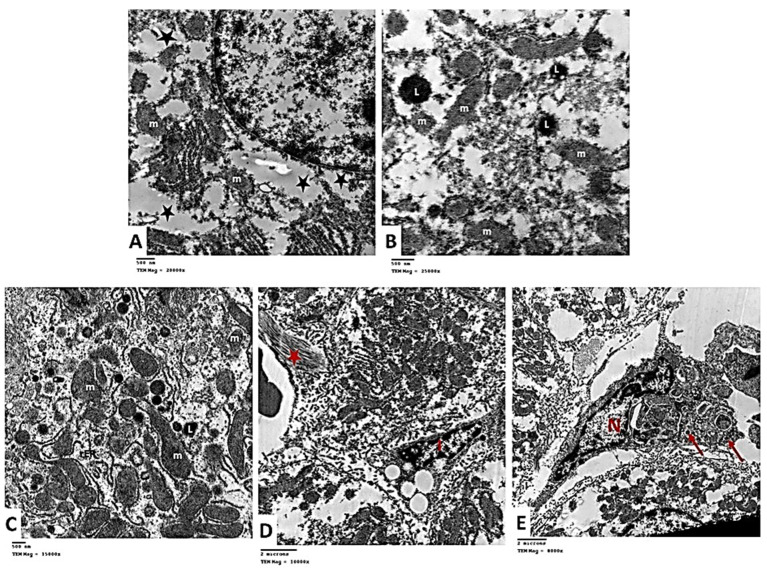
(**A**) Electron micrographs of Group II showing homogenous echogenic areas in hepatocyte cytoplasm (*). TEM × 20,000. (**B**) Electron micrograph showing the apparent decrease in rER and glycogen rosettes. Many lysosomes (L) are seen scattered in the cytoplasm. Mitochondria (m) are seen with an ill-defined outer membrane. Degeneration and lysis of the organelles are also noticed. TEM × 250,000. (**C**) showing swollen mitochondria (m), which lose their cristae. Expansion of rough endoplasmic reticulum (rER) is seen with loss of the attached ribosomes. TEM × 15,000. (**D**) Electron micrograph showing hepatic stellate cell or Ito cell (I) containing fat droplets in its cytoplasm. Notice the collagen fibers with their axial periodicity (*). TEM × 10,000. (**E**) Electron micrograph showing Kupffer cell with large irregular nucleus (N) and many micro-vesicles and phagocytosed material (↑) in the cytoplasm. TEM × 8000.

The ultra-structure of the hepatocytes in Group II showed destructive and degenerative changes. Homogenous echogenic areas were detected in the cytoplasm (Figure 12A). The apparent decrease in rER, and glycogen granules was noticed. Many lysosomes were seen scattered in the cytoplasm (Figure 12B). Some mitochondria appeared swollen, lost their cristae, and had an irregular outer membrane. Expansion of the rough endoplasmic reticulum was noticed with the loss of the attached ribosomes (Figure 12C). Hepatic stellate cells (HSCs, Ito cells) appeared with fat droplets in their cytoplasm. Nearby collagen fibers characterized by their axial periodicity were detected (Figure 12D). KCs were seen with many micro-vesicles and phagocytosed material in the cytoplasm (Figure 12E).

**Figure 13:**
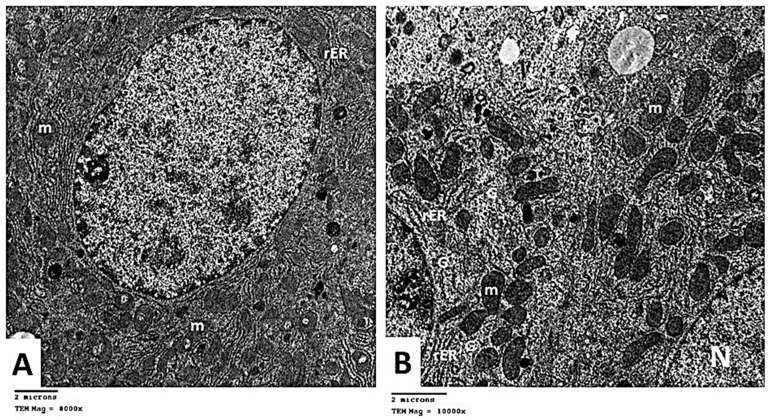
(**A**) Electron micrograph showing hepatocyte with nearly rounded nucleus (N) with mild irregularity in the nuclear membrane. Mitochondria (m) and rough endoplasmic reticulum (rER) are nearly similar to the control. Group III TEM × 8000. (**B**) Electron micrograph showing two hepatocytes. Part of the nucleus (N) is seen. Mitochondria of variable size and shape (m) are seen. An apparent increase in the rough endoplasmic reticulum (rER) and glycogen rosettes (G) is noticed compared to Group II. Group IV. TEM × 10,000.

Electron microscopic assessment of Group III hepatocytes demonstrated a nearly rounded nucleus with mild irregularity in the nuclear membrane. Mitochondria and the rough endoplasmic reticulum were nearly similar to the control (Figure 13A). In Group IV, the apparent increase in the rough endoplasmic reticulum and glycogen rosettes was noticed compared to Group II. Mitochondria was nearly similar to the control group (Figure 13B). These findings demonstrate that isoprenaline treatment causes various alterations in the ultra-structure of the liver tissue and that carvedilol administration could diminish these changes and restore the cells to a nearly normal state.

### 2.3. In Silico Molecular Docking Study

**Figure 14:**
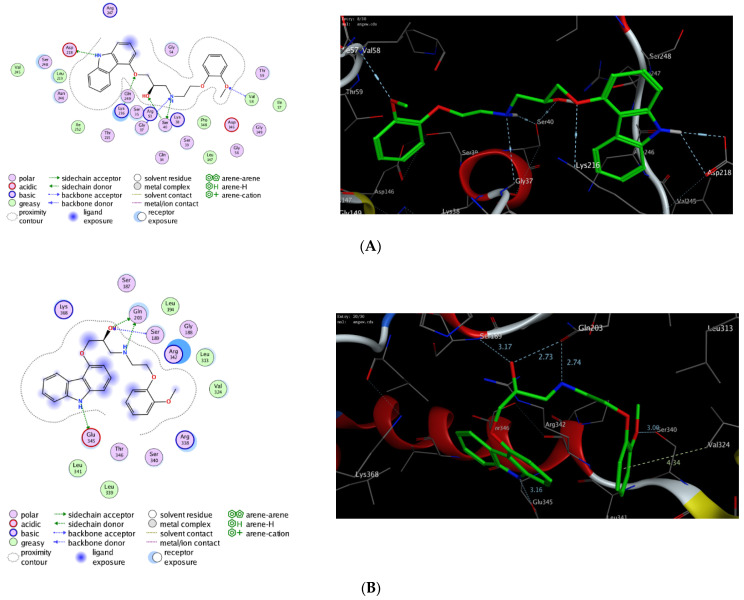
The (2D and 3D) molecular modelling interactions of carvedilol (green in 3D interactions) with dynamin-1-like protein (PDB code: *3w6p*) (**A**), and mitochondrial dynamics protein (PDB code: *4nxx*) (**B**). The hydrophobic interactions are shown as dotted blue arrows; (C atoms are colored grey, N blue and O red).

To further explore the mode of action of carvedilol, we have investigated the ability of carvedilol to target dynamin-1-like protein (DNM1L), and mitochondrial dynamics protein (MID51). Toward this aim, we have performed extensive molecular modelling studies to examine the binding affinity of carvedilol toward the binding site of DNM1L GTPase and MID51 proteins. On the PDB website, several 3D X-ray structures are available for the targeted mitochondrial proteins with different nucleotides [33,34,35,36,37,38]. In our study, we have selected the crystal structures (PDB codes) based on the resolution of crystallization and the ability of carvedilol to form (fully/partially) the main interaction of the original co-crystallized ligand. We first evaluated the docking method by redocking the original co-crystallized ligand to the binding pocket of the targeted proteins to ensure that the original ligand can bind similarly, as reported in the crystal structure, and forms the main interactions in the active site of the targeted protein. Next, the validated protocol was successfully used to evaluate the binding affinity of carvedilol toward the different targeted mitochondrial proteins (Table 5) [36,38]. The results showed that carvedilol has the ability to bind to the GTP binding sites of DNM1L and MID51 proteins with high binding affinity scores through a network of both hydrophilic and hydrophobic interactions (Figure 14). The binding of carvedilol toward DNM1L and MID51 proteins was thermodynamically favorable, as indicated by the negative values of the docking scores. Notably, carvedilol demonstrated the ability to bind to other amino acid residues in the binding pocket of DNM1L and MID51, compared to the essential ones in the crystallized form, and to develop extra hydrogen bonding. Among different investigated proteins, carvedilol demonstrated the best binding mode toward DNM1L protein with the highest binding affinity score. These results indicate that the protective activity of carvedilol could be attributed to its ability to bind to DNM1L protein.

## 3. Discussion

AHF is a severe public health problem that has a negative influence on people’s quality of life as well as shortening their longevity. The body’s capacity to satisfy the metabolic demands of skeletal muscles becomes progressively problematic as AHF advances [39]. Furthermore, AHF patients may develop liver-related symptoms that result in abnormal liver function due to diminished hepatic perfusion or as a side effect of drug toxicity [5]. Due to systemic problems and diseases that impact both the liver and the heart, AHF and liver disease frequently coincide, indicating that AHF can induce liver disease. Ischemic perfusion damage is defined as hypoperfusion-induced hypoxia that causes cell injury [40]. Hepatic ischemia–reperfusion injury in AHF is characterized by the stimulation of Kupffer cells, neutrophilic hepatitis activation, oxidative stress, damage to the mitochondria, and disruption of liver microcirculation [5]. In the present study, we aimed to analyze the biochemical profiles and histopathological findings of hepatic dysfunction in AHF, investigate the potential role of liver injury markers, and explore the possible strategy, which might assist in understanding cardio-hepatic interrelations, to effectively treat liver dysfunction in AHF. Furthermore, we shed light on the molecular process of ischemic hepatitis by examining mitochondrial fission and fusion dysfunction, a unique concept that might play a role.

The pathophysiological abnormalities in the heart muscle of Wistar rats are associated with those reported in human myocardial infarction [41]. Thus, isoprenaline-induced myocardial ischemia serves as a well-standardized model [42]. Isoprenaline is a non-selective stimulant of β-adrenergic receptors and is used to treat moderate and temporary heart blocks [43]. However, isoprenaline leads to myocardial infarction by dysregulating the oxygen supply to the heart, leading to inflammatory responses including the release of various cytokines [42,44]. Administration of isoprenaline causes an increase in ROS generation that leads to oxidative stress, oxidative damage, and degenerative abnormalities in the liver tissue [42,43,45,46]. Hepatic hypoperfusion causes damage to the integrity of the hepatic cell membrane, releasing liver enzymes into the bloodstream. Liver enzymes are commonly employed as indicators of liver failure and a better predictor of cardiovascular risk and oxidative stress. In our study, two doses of isoprenaline were administrated to induce AHF. It was observed that isoprenaline administration caused a considerable increase in the activities of liver enzymes (AST, ALT, ALP) and an increase in serum liver levels (total and direct bilirubin). Furthermore, isoprenaline treatment caused a significant reduction in serum albumin levels compared to the control group. Our findings are in agreement with the previously reported studies, which showed that isoprenaline-induced heart failure causes oxidative stress and reduces the blood flow to the liver, resulting in liver injury [42,45,46].

The liver is responsible for numerous functions, including the production and metabolism of a wide range of substances, as well as the detoxification of blood from harmful compounds [47]. It is the principal organ engaged in the biotransformation of xenobiotics by biochemical actions such as oxidation, reduction, or conjugation, creating ROS. As a result, in line with previous findings, the liver is particularly vulnerable to oxidative stress, which might explain the reduction in the hepatic SOD activity observed in our investigations in the isoprenaline-treated group [42,47,48]. MAPKs are a family of proteins that control various cellular responses, including cell proliferation, expression of genes, differentiation, mitotic division, and apoptosis [6,12,13,14]. Isoprenaline-induced ROS production caused an increase in the expression of MAPKs that allows cells to adapt to the stressful environment caused by ROS, therefore regulating cell function [43,49,50,51]. In accordance with previous studies, our results revealed an upregulation in the expression of the *MAPK* genes in the isoprenaline-treated group [43,49,52].

PGC-1α is the main mitochondrial biogenesis and function regulator, which modulates the increase in the ROS-induced oxidative stress via oxidative phosphorylation and ROS detoxification [53]. PGC-1α regulates oxidative metabolism either by cellular remodeling, via mitochondrial biogenesis, or by organelle remodeling via alterations in intrinsic mitochondrial characteristics [54]. PGC-1α regulates ROS elimination by increasing the production of multiple ROS-detoxifying enzymes, which affects the antioxidant defense system of the mitochondria and prevents ROS-induced cytotoxicity [55,56]. A decrease in PGC-1α expression due to redox disorder causes a metabolic syndrome that may lead to liver injury and dysfunction [21]. In agreement with previous reports, our investigations revealed that the hepatic PGC-1α expression attenuates in the isoprenaline-treated group, accompanied by high ROS levels [57,58,59].

Mitochondrial dynamics are modulated by the equilibrium between mitochondrial fission and fusion activities, and any abnormality can be associated with several diseases [22,60,61]. This dynamic equilibrium is regulated by membranal proteins, such as mitofusin 2 (Mfn2), and dynamin-1-like protein (DNM1L) [60,61,62]. Mfn2 modulates the fusion of the outer mitochondrial membrane, which extends protection against apoptotic stimuli. Accordingly, its role might be a cell defense mechanism against oxidative damage [63,64]. Our present data revealed a decrease in the Mfn2 expression after isoprenaline administration. These results indicate that the damage in the mitochondria of the hepatocytes occurs by the isoprenaline treatment. Several studies showed that Mfn2 is significantly suppressed in hepatic ischemia–reperfusion injury, which is in accordance with our results [65,66]. DNM1L regulates the fission of the mitochondrial membrane, an early apoptotic event [22,60]. In our investigations, the isoprenaline-treated group showed an upregulation in the expression of the mitochondrial fission protein DNM1L. These results indicate that hepatic ischemia associated with isoprenaline-induced AHF causes an imbalance in the mitochondria dynamics. Our results were in accordance with other studies that demonstrated that the increase in the DNM1L expression after isoprenaline administration is due to injury in the mitochondria [63,67].

MicroRNAs are short, non-protein-coding, single-stranded RNA sequences that control the expression of genes. In our investigations, we utilized miRDB-microRNA target prediction and functional study database, and microRNA Org target and expression database to define the list of regulatory miRNAs in ranking order. In this regard, mitofusin 2 and MAPKs were retrieved, as associated genes playing a role in mitochondrial dynamics modulation, from the gene atlas database and were used as inputs in our analysis. The results revealed that the pathway enrichment analysis of selected miRNA-17 has a higher number of target genes by using DIANA-mirPath software. MiRNA-17 is one of the proinflammatory miRNAs, which is recounted to initiate fibrosis development. In our study, an increase in the expression of hepatic miRNA-17 was observed in the isoprenaline-treated group, indicating that AHF may cause liver fibrosis [68]. Our findings are in accordance with previous studies, which reported that miRNA-17 is highly expressed in liver fibrosis, and could be a characteristic biomarker to evaluate liver fibrosis [68,69,70]. In our investigations, these results were further demonstrated by Mallory’s trichrome stain, which estimates the degree of deposition of hepatic fibrous tissue due to liver impairment. Our data revealed that, in the isoprenaline-treated group, the quantity of collagen fibers is increased around the central vein and in the portal area. Our findings were further confirmed by investigations of liver H&E-stained sections in the isoprenaline-treated group. Our results demonstrated the disruption of the normal hepatic architecture. Hemorrhage was also noticed in the hepatic lobules, blood sinusoids appeared dilated and congested in some areas, portal tracts were dilated, portal vein branches were congested, and bile ducts proliferated. Moreover, the ultra-structure of the hepatocytes showed destructive and degenerative changes, with an apparent decrease in rER. Some mitochondria appeared swollen, lost their cristae, and had irregular outer membranes. PCNA is an endogenous nuclear protein employed to recognize replicating cells in mammalian tissues [71]. PCNA antigen–antibody complexes display various staining patterns, categorized depending on the product’s location and intensity [72]. In our analysis, most of hepatocyte nuclei of the isoprenaline-treated group showed a negative reaction, indicating that AHF-induced hepatic ischemia causes hepatocyte degeneration. Taken together, these findings reveal that AHF-induced hepatic ischemia causes impairment in the hepatocytes.

**Figure 15:**
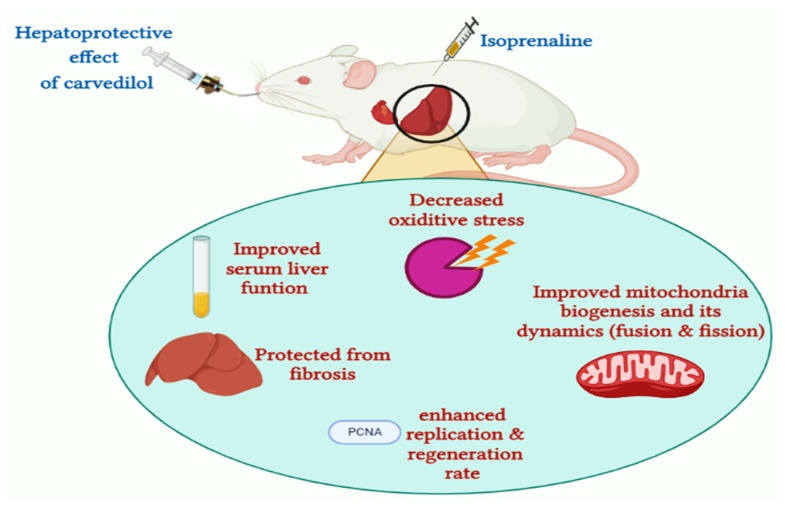
Hepatoprotective effect of carvedilol in ischemic hepatitis associated with acute heart failure.

β-blockers are a class of drugs used in the treatment of patients with cardiovascular disease [73]. They protect the ischemic myocardium by increasing coronary blood flow and thus helping the re-organization of blood flow to ischemic areas, which reduces the microvascular damage and inhibits platelet aggregation [32,74,75]. Among β-blockers, carvedilol is a non-selective β-adrenergic blocker, used for treatment of hypertension, left ventricular dysfunction, and heart failure [76,77]. Carvedilol has antioxidant effects and can improve myocardial function, increase survival, and decrease mortality in congestive heart failure [78]. Moreover, carvedilol decreases the infiltration of neutrophils, inhibits apoptosis, and helps to treat atherosclerotic disease formation and progression [29,76]. These multiple modes of action suggest that carvedilol may be used in the treatment of AHF-induced ischemic hepatic perfusion. Toward this aim, we examined the efficacy of carvedilol to pre- and/or post-treat ischemic hepatic perfusion induced by AHF. In our investigations, treatment with carvedilol (30 mg/kg) significantly improved liver functions, as compared to the isoprenaline-treated group. Indeed, the administration of carvedilol significantly decreased the activity of liver enzymes and the level of bilirubin, while increased albumin levels were observed in the control group. These results may be attributed to the enhancement of blood flow in the ischemic areas, which decreases liver injury caused by ischemia. Moreover, pre- or post-administration of carvedilol demonstrated an increase in the activity of the hepatic SOD enzyme and a decrease in hepatic MAPK compared to the isoprenaline-treated group. These results could be attributed to the antioxidant activity of carvedilol, which prevents free radical-induced liver damage. Our results are in accordance with the previous reports, which showed that the antioxidant effect of carvedilol originates from the carbazole moiety in its structure, and inhibits the free radical damage in chronic heart failure [74,79]. In addition, Abreu et al. stated that carvedilol inhibits mitochondrial dysfunction [80]. The effect of carvedilol on mitochondrial fission and fusion reversed the dysregulation process that occurred due to the administration of isoprenaline and decreased the hepatic hypoperfusion injury in cardiac patients. In our analysis, administration of carvedilol resulted in an increase in expression of hepatic Mfn2 and decrease in hepatic DNM1L. In addition, carvedilol increased the expression of PGC-1α and, hence, improved mitochondrial biogenesis. These results indicate that carvedilol has the ability to reverse the dysregulation in mitochondrial fission and fusion dynamics caused by isoprenaline-induced AHF to the normal state, and to decrease the hepatic hypoperfusion injury. Our results are in agreement with Wang et al. and Yao et al. who showed the permissive effect of carvedilol on Mfn2 and PGC-1α expression and mitochondrial biogenesis (Figure 15) [81,82].

Our present study demonstrated the downregulation of miRNA-17 and reduction in the amount of depositing collagen fibers in the Mallory’s trichrome stain in the group pre- and/or post-treated with carvedilol. These results reveal that the hepatoprotective effect of carvedilol could be attributed to its ability to diminish the fibrous tissue deposition, which reduces hepatic cell damage and necrosis. Our results were in accordance with previously reported studies, which found that administration of carvedilol diminishes the development of hepatic fibrosis [83,84,85]. Our histological findings revealed an apparent increase in the rough endoplasmic reticulum and glycogen rosettes, and the mitochondria appeared, to some extent, as that of the control group. The electron microscopic examination of hepatocytes showed nearly three rounded nuclei with mild irregularity in their nuclear membranes. The mitochondria and rough endoplasmic reticulum appeared similar to that in the control group. The histological effects of carvedilol on the liver tissues were previously discussed in different animal models and are in agreement with our results [31,84]. In the current study, carvedilol significantly increased the mean area percentage of the PCNA-positive immune reaction and the mean number of PCNA-positive nuclei. Together, these results demonstrate the ameliorative effect of carvedilol on hepatocyte degeneration by enhancing their replication and regeneration rate after AHF-induced hepatic ischemia. To the best of our knowledge, this the first report for the protective effects of carvedilol on ischemic hepatic perfusion associated with AHF. It should be noted that this study has some limitations, including the small sample size, the application of the non-drug model of AHF, and further investigation being needed to affirm the current findings. Further, we were unable to account for all available variables to investigate the potential hepatoprotective effects of carvedilol. Nevertheless, our findings provide potential information about the targeting of the epigenetic regulator mi-RNA17 and the hepatoprotective role of carvedilol.

To further explore the mode of action of carvedilol, we have performed a molecular modelling study to investigate the binding affinity of carvedilol toward the binding site of different mitochondrial proteins, mainly DNM1L and MID51, that mediate mitochondrial morphology. Indeed, the morphology of mitochondria is dynamically regulated by an equilibrium between fusion and fission [86]. DNM1L is a mitochondrial protein that mediates the mitochondrial membrane fusion through a GTPase-dependent mechanism which includes its oligomerization around a scission site, a step regulated by several membrane proteins [35,87]. DNM1L plays a vital role in endocytic vesicle formation and normal development of the brain, including the cerebellum [88,89,90]. Further, DNM1L meditates programmed apoptosis and necrosis during normal neural tube formation [91,92]. On the other hand, MID51 is a mitochondrial membrane protein that mediates mitochondrial fission [38,93,94]. MID51 mediates the GTPase activity and oligomerization of DNM1L protein by promoting the association of DNM1L protein to the mitochondrial surface. MID51 can bind to ADP and GDP with lower affinity, but not to GTP, UDP, ATP, CDP, and AMP. Although the presence of bound ADP is required to promote the GTPase activity of DNM1L, MID51 does not require bound ADP to recruit DNM1L [38,95,96,97]. In the present study, we have deeply investigated the potency of carvedilol to bind to the active site of DNM1L and MID51 proteins. As indicated in Table 3, carvedilol exhibited high to moderate binding affinity scores toward the binding pocket of DNM1L and MID51 proteins. Toward DNM1L, carvedilol showed the ability to bind to Lys216 and Val58 through the phenolic hydroxyl groups, while the carbazole amino group could bind to the Asp218 amino acid residue. Further, the Ser40 amino acid residue formed two hydrogen-bonding interactions with the secondary hydroxyl group and the secondary amino group of the scaffold. The secondary amino group could also bind to the Gly37 residue (Figure 10). The binding pose of carvedilol was also supported by a set of hydrophobic interactions with greasy amino acid residues in the binding pocket of DNM1L protein (Ile57, Val58, Leu147, Pro148, Leu219, Ile252, Val245). On the other hand, carvedilol could bind to the MID51 binding pocket by forming only four hydrogen-bonding interactions with Gln203, Ser189, Glu345, Val324, and Ser340 amino acid residues via the carbazole amino group, phenolic hydroxyl, and the secondary hydroxyl and amino groups. Further, carvedilol showed the ability to form other hydrophobic interactions with Leu194, Leu313, Val324, Leu339, and Leu341 amino acid residues (Figure 10). Taken together, these results indicate that carvedilol has a high potency to bind to DNM1L protein, and that the protective activity of carvedilol could be attributed to its ability to target this protein. Further studies should be conducted in the future to confirm the potency of carvedilol toward DNM1L and MID51 proteins

## 4. Materials and Methods

### 4.1. Chemicals and Reagents

Pentobarbital sodium salt (product number: 5178), isoprenaline hydrochloride (product number: I5627) and *carvedilol* (product number: C3993) were obtained from Sigma-Aldrich Darmstadt, Germany.

### 4.2. Groups and Animals

The research and ethical advisory board of the Ain Shams University Faculty of Medicine examined and approved the whole experimental procedure. (No. FWA 00027031, 11/2021), and the greatest care was given throughout the experimental procedure, including during the sacrifice in accordance with the rules of the 8th edition, National Academies Press. Twenty-four male Wistar rats (their weights 150–200 g) obtained from the Institute of National Research Center (Cairo, Egypt) were kept in an animal house that kept temperature (20 ± 2 °C) and 12 h daylight cycle regulation. Before beginning the experimental technique, a week of acclimatization was allowed. The rats then were divided into four groups at random:

Group I: The naïve group (*n* = 6): received 1 mL of saline two times (s.c., 24 h apart).

Group II: Control positive group (*n* = 6): isoprenaline was administered two times to Wistar rats (170 mg/kg, s.c., 24 h apart) [98].

Group III: Pre-treated group (*n* = 6): carvedilol dissolved in distilled water (30 mg/kg/day, PO) was administrated two weeks before isoprenaline administration and the rats were immediately sacrificed [99].

Group IV: Post-treated group (*n* = 6): carvedilol was administrated (30 mg/kg/day, PO) just after isoprenaline administration for two weeks until they were sacrificed.

### 4.3. Induction of Hepatic Hypoperfusion

Acute heart failure (AHF) was prompted by the administration of isoprenaline in two distinct doses to male Wistar rats (170 mg/kg, s.c.), doses administered 24 h apart [98].

### 4.4. Samples Processing

1. Blood samples

After two weeks, rats from each group were anaesthetized with 3% pentobarbital sodium, and we took blood samples from the retro-orbital blood vessels. Then, rats were sacrificed by exsanguination. Serum was isolated from whole blood taken and stored at −80 °C until it was needed.

2. Liver tissue samples

The liver was sliced and thoroughly cleansed with ice cold saline. Following homogenization of the liver in phosphate-buffered saline known as PBS with its pH adjusted to 7.4, we centrifuged the supernatants and then stored it at −80 °C until testing. For histological investigation, an additional part of the liver was taken and kept in 10% neutral buffered formalin solution.

### 4.5. Biochemical Assays

#### 4.5.1. Evaluation of Liver Function

Liver function was assessed by evaluating the activity of transaminases enzymes (AST (product number: MAK055), ALT (product number: MAK052)), alkaline phosphatase enzyme (ALP) (product number: MAK447), and the level of total and direct bilirubin (product number: MAK126), and albumin (product number: MAK125) in serum, using endpoint colorimetric assay kits obtained from Sigma-Aldrich Darmstadt, Germany.

#### 4.5.2. Assessment of the Activity of Hepatic Superoxide Dismutase Enzyme (SOD)

The enzyme-linked immunosorbent assay (ELISA) was employed to detect the activity of SOD in liver tissue lysate according to the prescribed standards (Cat. No. MBS266897, My BioSource, San Diego, CA, USA). After 10 min, the intensity of the color in each well was determined by means of a microplate reader set to 450 nm (with the reference absorbance at 540 nm).

#### 4.5.3. Isolation of miRNA-17

We retrieved miRNA-17 regulating many target genes involved in liver injury through MicroRNA target prediction and functional study database (available at http://www.mirdb.org/, accessed on 1 October 2021), and microRNA Org target and expression database (available at http://www.microrna.org/microrna/home.do, accessed on 1 October 2021). The current bioinformatics analysis was focused on specific miRNA, which was published on liver ischemic-reperfusion

The miRNA-17 was selected as it is associated with apoptosis and liver cell injury. An RNeasy mini kit (Qiagen, Hilden, Germany) was utilized to isolate total RNA using rat liver tissue samples, according to the prescribed standards. A NanoDrop^TM^1000 Spectrophotometer was used to detect the concentration of RNA in each sample (Thermo Fisher Scientific, Waltham, MA, USA). A260/A280 absorbance ratios of 1.8–2.1 were widely recognized as “absolute” for RNA. Reverse transcription was performed according to the prescribed standards employing MiScript II RT PCR kits (Qiagen catalogue no. 218161, Hilden, Germany). Prior to real-time PCR, the reverse transcription processes were held at −20 °C. The RNeasy mini kit (Qiagen, Hilden, Germany) was employed to isolate total RNA from rat liver tissue samples, following the prescribed standards. To verify the RNA quantity in each sample, we used a NanoDrop^TM^1000 Spectrophotometer (Thermo Fisher Scientific, USA). A260/A280 absorbance ratios of 1.8–2.1 were widely recognized as “absolute” for RNA.

#### 4.5.4. Assessment of miRNA-17, MAPK, PGC 1α, Mitofusin 2, and Dnm1l Expression by qPCR

The miRNA-17, MAPK, PGC 1α, mitofusin 2, and DNM1L were measured by adding 10 µL 2× RT² SYBR Green ROX qPCR Master mix, then forward and reverse primer assay was undertaken for miR-17 5′-GCAGGAAAAAAGAGAACATCACC-3′ and 5′-TGGCTTCCCGAGGCAG-3′, MAPK 5′-GCTCCTTCGACGTGACCTTT-3′ and 5′-TCCAGTACCACGTAGACAGA-3′ PGC 1α 5′-CCC TGC CAT TGT AAA GAC-3′ and 5′-TGC TGC TGT TCC TGT TTT -3′, mitofusin 2 5′-GCC AGC TTC CTT GAA GAC AC-3′ and 5′-GCA GAA CTT TGT CCC AGA GC-3′ DNM1L 5′ GCGCTGATCCCGCGTCAT 3′ and 5′ CCGCACCCACTGTGTTGA 3, respectively. A total of 2 µL of template cDNA were added to RNase-free water to an end volume of 20 µL. An ACTB_1_SG QuantiTect Primer Assay (NM_001101) was utilized as the housekeeping gene designed to standardize our obtained data and evaluated with a reference sample. The PCR technique for relative measurement of miRNA-17, MAPK, PGC 1α, mitofusin 2, and DNM1L was as follows: 10 min of denaturation at 95°C; then, it went through 45 denaturation cycles at 95 °C for 15 s, 30 s of annealing at 55°C, and 30 s of extension. In each sample, the relative quantification of RNA was estimated using the Schmittgen and Livak technique. Each sample’s threshold cycle (Ct) estimate was determined using a Rotor Gene real-time PCR recognition apparatus (Qiagen, Hilden, Germany). The melting curve was examined to determine the amplicon’s characteristics for qPCR [100].

### 4.6. Histological Studies

#### 4.6.1. Light Microscopic Study

An abdominal incision was used to dissect the liver. Each specimen’s right lobe was placed in 10% formol saline for fixation. Then, it was dehydrated, cleaned, and paraffin-block mounted. Sections were cut at 5 mm intervals and were put in H&E and Mallory’s trichrome stains.

- Histopathological grading and scoring of liver changes (H&E stain):

Suzuki scoring standards were used to determine the amount of liver injury. None = 0, minimum = 1, slight = 2, moderate = 3, and extreme = 4 were assigned to congestion, cell swelling, and vacuolization. Liver necrosis was scored as follows: none = 0, necrosis of individual cell = 1, up to 30% lobular degeneration = 2, up to 60% lobular degeneration = 3, and more than 60% lobular degeneration = 4. (Table 6).

All the identified components were evaluated in four fields of the hepatic sections stained by H&E of 5 rats in each group with, overall, 20 microscopic fields in each group, via a Carl Zeiss (Berlin, Germany) microscope by an objective lens ×10 (low-power field) in the Histology Department, Faculty of Medicine, Ain Shams University.

#### 4.6.2. Immunohistochemical Study

Additional paraffin sections were cut on slides that were positively charged and tested for proliferating cell nuclear antigen (PCNA) immunity. The brown nuclear response indicated a positive reaction to the PCNA immune-histochemical approach. A portion of the skin’s epidermis was stained as a positive control. The PCNA kit was acquired from Lab Vision in California, USA, at a dilution of 1:200–400 for one and a half hours [101]. Sections were photographed using a Canon EOS 1100D Digital SLR camera and a Leica DM2500 microscope in the Histology and Cell Biology Department of the Faculty of Medicine at Ain Shams University. In addition, tiny sections of liver tissue were quickly preserved and processed for TEM analysis. A JEOL- 1010 Transmission electron microscope made in Japan was used to study and photograph specimens at the Regional Center of Mycology and Biotechnology, Azhar University, Cairo, Egypt.

#### 4.6.3. Morphometric Studies

The image analyzer program Leica Q win V.3 was used, which was downloaded onto a computer at the Ain Shams University, Faculty of Medicine, Department of Histology and Cell Biology. A Leica DM2500 microscope was linked to the computer (Wetzlar, Germany). Morphometric analysis was performed on specimens from all groups. Five different non-overlapping fields from five different sections of different rats were examined in each group for measuring each of the following:

1. The mean proportion of positive immune reactions of PCNA
2. The average number of nuclei exhibiting a significant immunological response to PCNA.
3. Mean area percentage of collagen fibers stained by Mallory’s trichrome

Data were gathered, reviewed, and statistically analyzed using one-way ANOVA in the SPSS.21 program (IBM Inc. Chicago, IL, USA). Then, we recorded our data as the mean ± standard deviation (SD). The significance of the data was assessed by the *p* value. The data were considered as non-significant if *p* values were > 0.05, while the data were considered as significant if *p* values were < 0.05.

#### 4.6.4. Transmission Electron Microscopic Study (TEM)

Small pieces of liver tissue were rapidly fixed and then processed for a transmission electron microscopic study (TEM). Specimens were examined and photographed with a JEOL- 1010 transmission electron microscope made in Japan at EM unit at the Regional Center of Mycology and Biotechnology, Azhar University, Cairo, Egypt.

### 4.7. In Silico Molecular Modelling Study

Carvedilol’s affinity for the active sites of dynamin-1-like protein (DNM1L), and mitochondrial dynamics protein (MID51) was investigated by a molecular docking study utilizing Molecular Operating Environment software (MOE, 2015.10). On the protein data bank (PDB), there are several 3D X-ray crystal structures for DNM1L (PDB codes: *3w6n*, *3w6o*, *3w6p*, *4h1u*, *4h1v*, *4bej*, and *5wp9*) [33,34,35,36], and MID51 (PDB codes: *4nxw*, *4nxx*, *4nxu*, *5x9b*, and *5x9c*) proteins [37,38]. The crystal structures (PDB codes) were selected based on the resolution of crystallization and the ability of carvedilol to bind in similar binding poses as the original co-crystallized ligand. Accordingly, the molecular docking study was performed for the following PDB codes; *4nxx* (MID51) and *3w6p* (DNM1L). The PDB website (http://www.rcsb.org/pdb, 1 April 2022) was used to retrieve the 3D crystalline structures of the targeted proteins. The 2D and 3D structures of carvedilol were attained using the ChemDraw professional program (cds.15.1) and Discovery Studio software, respectively. The 3D crystal structures were initially prepared for the docking process by removing the water molecules and ions, deleting the extra chains, and protonating the protein. The geometry of the protein was optimized by applying the MMFF94x force field and conf search module in the default mode in the MOE program. Next, the docking protocol was examined for validation by docking the original co-crystallized ligand/inhibitor to the binding pocket of the targeted protein by utilizing the London dG scoring function and Triangle Matcher placement method [102,103,104,105,106,107,108]. The validated protocol was then used to explore the binding affinity of carvedilol toward the active sites of the DNM1L and MID51 proteins. The acquired data was analyzed and evaluated to obtain the most stable binding poses with the highest binding affinity score.

### 4.8. Statistical Analysis

All values were expressed as mean ± S.E.M. Statistical analysis was performed utilizing the GraphPad prism software program (version 7.0 (2016) Inc., San Diego, CA, USA). Data was assessed for normality using the Shapiro–Wilk normality test. The statistical difference among groups was analyzed using one-way ANOVA followed by Tukey’s post-hoc test for comparison between groups. All *p* values < 0.05 were considered statistically significant. The sample size was defined using the GraphPad Stat Mate software program, (version 4, 2005). The sample size was calculated by the following analysis and data: analysis by comparing two means using the unpaired t-test; threshold *p*-value (Alpha) = 0.05, two-tailed Student t-test; anticipated SD of each group; the difference between means (effect size); and power of the experiment = 80%.

## 5. Conclusions

In the current study, we showed that isoprenaline-induced AHF leads to ischemia in hepatic perfusion, resulting in alterations in the biochemical, immunohistochemical, and histological characteristics of liver tissue. Our results revealed that AHF-induced hepatic ischemia caused a significant reduction in liver function, as indicated by evaluation of AST, ALT, and ALP activities, and the level of total and direct bilirubin, and serum albumin. Further, hepatic ischemia evaluated the expression of the oxidative stress biomarkers (MAPKs) and impaired the mitochondrial dynamics by downregulating the hepatic PGC-1α and Mtf2 expression, while increasing the expression of hepatic DNM1L, and their epigenetic regulator miRNA-17. We further explored the hepatoprotective effect of carvedilol and showed that it has the ability to improve all the biochemical, immunohistochemical, and histological parameters of liver tissue, and restore liver tissues to their normal state. Our investigations revealed that the hepatoprotective activity of carvedilol was as a result of its antioxidant activity, but also its ability to target the mitochondrial dynamics-related proteins and the epigenetic miRNA-17 expression. Based on our study, we hypothesize that early treatment with carvedilol for patients with AHF could promptly protect and ameliorate ischemia–reperfusion injury in liver tissues and hepatic damage. Further, we propose that the PGC-1α, Mtf2, DNM1L, and miRNA-17 pathways could be considered as early prognostic markers of hepatic damage and could allow monitoring of the healing process and drug response. To the best of our knowledge, this is the first report to examine the effect of the mitochondrial dynamics-related proteins (PGC-1α, Mtf2, and DNM1L) and the epigenetic regulator, miRNA-17, pathways in targeting hepatic ischemia, and to explore the hepatoprotective role of carvedilol in regulating this pathway.

## Figures and Tables

**Table 1:** Effect of carvedilol on liver function in hepatic ischemia associated with isoprenaline-induced AHF.

Parameters	Group I	Group II	Group III	Group IV
Serum AST(IU/L)	32 ± 1.27	123.75 ± 8.65 ^a^	47.75 ± 2.62 ^ab^	73 ± 5.29 ^abc^
Serum ALT(IU/L)	30.5 ± 2.4	92 ± 3.9 ^a^	45.25 ± 3.90 ^ab^	55.25 ± 4.03 ^abc^
AST/ALT	1.07 ± 0.22	1.35 ± 0.19 ^a^	1.05 ± 0.11 ^ab^	1.32 ± 0.09 ^abc^
ALP (IU/L)	111.3 ± 3.88	322 ± 7.82 ^a^	123 ± 14.58 ^ab^	151.3 ± 5.5 ^abc^
Total bilirubin (mg/dL)	0.55 ± 0.12	10.4 ± 0.32 ^a^	1.62 ± 0.15 ^ab^	2.77 ± 0.15 ^abc^
Direct bilirubin (mg/dL)	0.12 ± 0.23	8.9 ± 0.01 ^a^	1.7 ± 0.32 ^ab^	2.6 ± 0.38 ^abc^
Albumin (g/dL)	5.18 ± 0.25	2.43 ± 0.16 ^a^	4.1 ± 0.08 ^ab^	3.8 ± 0.7 ^abc^

Group I: naïve, Group II: control positive, Group III: carvedilol pre-treated, Group IV: carvedilol post-treated. Data shown as mean ± SEM. ^a^ *p* < 0.05 versus group I, ^b^ *p* < 0.05 versus group II, ^c^ *p* < 0.05 versus group III.

**Table 2:** Effect of carvedilol on number and frequency distribution (%) of each component examined in hepatic ischemia associated with isoprenaline-induced AHF.

Groups	Group I	Group II	Group III	Group IV
Congestion 0: None	18 (90%)	3 (15%)	15(83%)	10 (50%)
1: Minimum	2 (10%)	10 (50%)	3 (17%)	7 (35%)
2: Slight	0	6 (30%)	0	3 (15%)
3: Moderate 4: Extreme	0 0	1 (5%) 0	0 0	0 0
Vacuolization 0: None	19 (95%)	12 (67%)	18 (90%)	16 (80%)
1: Minimum	1 (5%)	6(33%)	2(10%)	4 (20%)
2: Slight	0	0	0	0
3: Moderate 4: Extreme	0 0	0 0	0 0	0 0
Necrosis 0: None	20 (100%)	2 (10%)	19 (95%)	13 (65%)
1: Necrosis of individual cell	0	5 (25%)	1 (5%)	5 (25%)
2: <30%	0	13 (65%)	0	2 (10%)
3: 30–60% 4: >60%	0 0	0 0	0 0	0 0

**Table 3:** Effect of carvedilol on % area of collagen fibers in liver sections of different groups in hepatic ischemia associated with isoprenaline-induced AHF.

Groups	Group I	Group II	Group III	Group IV
Area % of collagen	1.63 ± 0.26	6.41 ± 1.11 ^a^	2.33 ± 0.60 ^ab^	5.16 ± 0.93 ^abc^

Group I: naïve, Group II: control positive, Group III: carvedilol pre-treated, Group IV: carvedilol post-treated. Data are presented as mean ± SEM, ^a^ *p* < 0.05 versus group I, ^b^ *p* < 0.05 vs. control group II, ^c^ *p* < 0.05 vs. group III.

**Table 4:** Effect of carvedilol on the mean area percentage of PCNA immune reaction and the mean number of PCNA positive nuclei.

	Mean Area Percentage of PCNA	Mean Number of PCNA Positive Nuclei
Group I	2.19 ± 0.35	51.85 ± 1.06
Group II	1.50 ± 0.57 ^a^	49.28 ± 0.95 ^a^
Group III	11.95 ± 0.82 ^ab^	130.83 ± 1.47 ^ab^
Group IV	13.43 ± 0.42 ^abc^	133.42 ± 1.51 ^abc^

Group I: naïve, Group II: control positive, Group III: carvedilol pre-treated, Group IV: carvedilol post-treated. Data are presented as mean ± SEM. ^a^ *p* < 0.05 versus group I, ^b^ *p* < 0.05 vs. control positive, ^c^ *p* < 0.05 vs. carvedilol pre-treated groups.

**Table 5:** Binding scores and interactive residues of carvedilol in the binding pocket of dynamin-1-like protein (DNM1L) and mitochondrial dynamics protein (MID51).

Protein	PDB Code	Docking Score (kcal/mol)	Interactive Residues	
			Hydrophilic Interactions	Hydrophobic Interactions
Dynamin-1-like protein (DNM1L)	3w6p	−14.83	Val58, Ser40, Gly37, Lys216, Asp218	Ile57, Val58, Leu147, Pro148, Leu219, Ile252, Val245
Mitochondrial dynamics protein (MID51)	4nxx	−12.69	Gln203, Ser189, Glu345, Val324, Ser340	Leu194, Leu313, Val324, Leu339, Leu341

**Table 6:** Characteristics of liver ischemia–reperfusion injuries.

Score	Congestion	Vacuolization	Necrosis
0	None	None	None
1	Minimum	Minimum	Necrosis of individual cell
2	Slight	Slight	< 30%
3	Moderate	Moderate	30–60%
4	Extreme	Extreme	>60%

## Data Availability

Data is contained within the article.

## Abbreviations

AHF: acute heart failure, ALP: alkaline phosphatase, ALT: alanine aminotransferase, AST: aspartate aminotransferase, CV: central vein, DNM1L: dynamin-1-like protein, KC: Kupffer cell, MAPK: mitogen-activated protein kinase, MID51: mitochondrial dynamics protein, miRNA: microRNA, Mtf: Mitofusin, PCNA: proliferating cell nuclear antigen, PGC-1α: peroxisome proliferator-activated receptor co-activator 1α, rER: rough endoplasmic reticulum, ROS: reactive oxygen species, SOD: superoxide dismutase, STAT3: signal transducer and activator of transcription 3, and TEM: transmission electron microscopic.
